# Size-specific recolonization success by coral-dwelling damselfishes moderates resilience to habitat loss

**DOI:** 10.1038/s41598-020-73979-0

**Published:** 2020-10-12

**Authors:** Morgan S. Pratchett, Vanessa Messmer, Shaun K. Wilson

**Affiliations:** 1 ARC Centre of Excellence for Coral Reef Studies, James Cook University, Townsville, QLD 4811 Australia; 2 Marine Science Program, Department of Biodiversity, Conservation and Attractions, Western Australian Government, Kensington, WA 6151 Australia; 3 Oceans Institute, University of Western Australia, Crawley, WA 6009 Australia

**Keywords:** Behavioural ecology, Community ecology

## Abstract

Increasing degradation of coral reef ecosystems and specifically, loss of corals is causing significant and widespread declines in the abundance of coral reef fishes, but the proximate cause(s) of these declines are largely unknown. Here, we examine specific responses to host coral mortality for three species of coral-dwelling damselfishes (*Dascyllus aruanus*, *D. reticulatus*, and *Pomacentrus moluccensis*), explicitly testing whether these fishes can successfully move and recolonize nearby coral hosts. Responses of fishes to localized coral loss was studied during population irruptions of coral feeding crown-of-thorns starfish, where starfish consumed 29 (34%) out of 85 coral colonies, of which 25 (86%) were occupied by coral-dwelling damselfishes. Damselfishes were not tagged or individually recognizable, but changes in the colonization of different coral hosts was assessed by carefully assessing the number and size of fishes on every available coral colony. Most damselfishes (> 90%) vacated dead coral hosts within 5 days, and either disappeared entirely (presumed dead) or relocated to nearby coral hosts. Displaced fishes only ever colonized corals already occupied by other coral-dwelling damselfishes (mostly conspecifics) and colonization success was strongly size-dependent. Despite movement of damselfishes to surviving corals, the local abundance of coral-dependent damselfishes declined in approximate accordance with the proportional loss of coral habitat. These results suggest that even if alternative coral hosts are locally abundant, there are significant biological constraints on movement of coral-dwelling damselfishes and recolonization of alternative coral habitats, such that localized persistence of habitat patches during moderate or patchy disturbances do not necessarily provide resilience against overall habitat loss.

## Introduction

Many species exist as metapopulations (or metagroups) occupying fragmented patches of suitable habitat^1^. Declines in the quantity or quality of habitat patches, as well as increasing distances among habitat patches (habitat fragmentation), can all have significant effects on the local abundance and persistence of habitat-associated species^1,2^. Local persistence of such species is conditional upon recolonization of vacant habitat patches and/or movement between habitat patches in accordance with changes in habitat condition^3,4^. On coral reefs, many fishes occupy discrete coral colonies^5–7^, and the composition, size and abundance of specific coral hosts exert major constraints on local and geographic abundances of these fishes^5,8–10^. Declines in the abundance and composition of coral habitats following acute disturbances invariably lead to rapid and pronounced declines in the local abundance of coral-dwelling reef fishes, especially for species with very specific resource requirements^11–15^.

Coral reefs are currently subject to unprecedented levels of disturbance^16–19^, causing sustained and ongoing declines in the abundance of habitat-forming corals. Causes of coral loss vary regionally, and are increasingly being compounded by anthropogenic climate change^20–22^. One of the major contributors to sustained declines in coral cover in the Indo west-Pacific are population irruptions of coral feeding crown-of-thorns starfish (CoTS), *Acanthaster* spp.^19,23–25^. Aside from causing extensive and widespread coral depletion^24,26^, *Acanthaster* spp. feed disproportionately on certain coral types and can have strong selective effects on the structure of coral assemblages^27,28^.

Crown-of-thorns starfish have inherent feeding preferences^29^, but also avoid certain corals because they are defended by coral-associated organisms. Xanthid crabs, and mainly *Trapezia* spp., are the predominant organisms implicated in defending corals from CoTS^30–32^. However, the effectiveness of these crustacean guards may be enhanced by activities of coral-associated fishes^33,34^. Weber and Woodhead^33^ report seeing *Dascyllus aruanus* bite the leading tube feet of a CoTS in Fiji, which together with activities of crabs inhabiting the same coral, ultimately prevented CoTS from feeding on their host colony of *Stylophora*. Several species of territorial damselfishes (e.g., *Plectroglyphidodon dicki and Stegastes nigricans*) have also been observed to attack and effectively repel CoTS^34,35^. Coral-dwelling damselfishes are known to feed on larval CoTS potentially regulating the local abundance of these organisms^36,37^, but may also play a role in deterring adult CoTS from feeding on their host colonies. If not, responses of coral-dependent fishes to coral loss caused by *Acanthaster* spp. (or any other disturbances) will depend on the specific overlap in patterns of habitat use versus habitat vulnerability^12,15,38^.

Many studies have explored the effects of coral loss on coral reef fishes^39–41^, focusing mainly on changes in the abundance or diversity of fishes following large-scale disturbances that cause extensive coral loss. While increasing environmental changes that are emerging as predominant cause of coral loss^22^ also have direct effects on coral reef fishes^42–44^, there has been little research to date on the specific behavioral responses of fishes to acute coral loss^45,46^. It is implicitly assumed, for example, that declines in the abundance of reef fishes following extensive and widespread coral loss are due to elevated rates of individual mortality, linked to resource depletion and loss of individual condition and/or increased susceptibility to predation^40,47,48^, combined with reduced levels of population replenishment^49,50^. Coker et al.^51^ showed that coral-associated mesopredators (*Pseudochromis fuscus*) were almost twice as likely to strike at potential prey fishes associated with the stark white bleached or recently dead corals compared to equivalent prey on strongly pigmented, unbleached, corals. Even if predation does not cause increased in situ mortality, it is likely that increased exposure to predators will provide significant motivation for coral-dwelling fishes to rapidly vacate bleached and/or dead coral hosts^47,51^.

Coral-dwelling damselfishes (family Pomacentridae) comprise a diverse and numerically dominant assemblage of fish species that are often found living in close association with live coral colonies^52,53^. Damselfishes from the genera *Dascyllus* and *Pomacentrus* tend to occupy all-purpose home ranges or territories within the immediate vicinity of a single branching coral colony^7,54–56^. Whilst these damselfishes are critically dependent on their host corals^12,15^, the proximate causes of declines in their abundance following host coral mortality, remain largely unexplored. Of particular interest is timing of responses relative to changes in the physical and biological structure of host corals^40,47,57^. Feary et al.^49^ suggested that coral-dwelling fishes vacate host corals as soon as they bleach, let alone die. However, several other studies have recorded persistent associations between coral-dwelling damselfishes and their coral hosts even after coral bleach or die^58–60^. It is also unclear whether the disappearance of fishes from specific host corals necessarily represents mortality, or movement of individuals to new and alternative coral habitats. Knowledge of the capacity of site-attached fishes to relocate and colonize alternative habitats following habitat perturbations, and the identification of social, ecological and physical impediments to such recolonization^45^, is central to understanding patch dynamics and metapopulation resilience of fishes with strong microhabitat associations.

The aims of this study were firstly, to test whether coral colonies occupied by coral-dwelling damselfishes are more or less likely to be consumed by the Pacific CoTS (*Acanthaster* cf. *solaris*). Previous research has shown that coral infauna (particularly, coral crabs) may be effective in deterring *A.* cf. *solaris* from feeding on their host corals^30–32^, but it is unknown whether coral-dwelling damselfishes effectively defend host corals. Secondly, we examined the specific responses of damselfishes following host coral mortality, using intensive sampling to assess if and when these fishes vacate dead coral hosts. Specifically, we recorded the number and individual size of damselfishes that resided within all possible coral hosts to infer patterns of relocation, and test whether coral-dwelling damselfishes are generally resilient to host coral mortality given availability of alternative coral hosts within the immediate vicinity. This study is important in understanding the responses of site-attached fishes during moderate or patchy disturbances, whereby at least some corals persist^13,61,62^. However, it is unknown to what extent the responses of coral-dwelling fishes is moderated by the capacity of displaced fishes to colonize new and alternative coral habitats^45,58^. If so, we might expect an increase in the densities of damselfishes within surviving coral colonies or increasing use of previously unoccupied (and presumably therefore sub-optimal) coral hosts with no net change in the local densities of coral-dwelling damsels.

## Results

**Figure 1:**
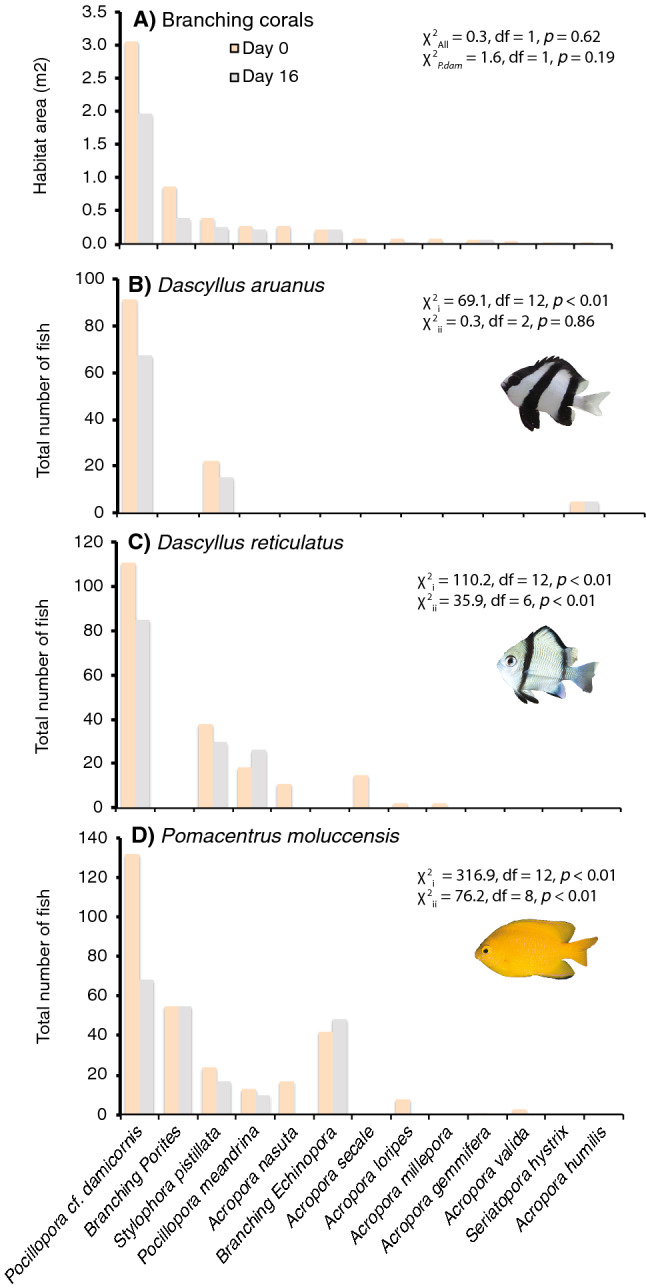
Changes in availability and use of different branching corals across the four (10 × 10 m) experimental plots from day 0 (orange) to day 16 (grey). Habitat availability (**A**) is calculated based on the sum of estimated projected areas of live tissue for every colony of each coral taxa (species or morphotype). χ^2^ statistics (Pearson statistic with Yate’s correction) were used to compare the proportion of corals that were consumed by CoTS relative to occupation by damselfishes for all corals combined (χ^2^_All_) and then for *P.* cf. *damicornis* separately (χ^2^_Pdam_). Changes in habitat use for *Dascyllas aruanus* (**B**), *Dascyllus reticulatus* (**C**), and *Pomacentrus moluccensis* (**D**) are shown based on the total number of fishes recorded across all colonies of each coral taxa. Here, χ^2^_i_ compares patterns of coral use by each damselfish species on day 0 with proportional availability of the different corals, and χ^2^_ii_ tests for changes in patterns of habitat use between day 0 and day 16.

A total of 85 distinct colonies, from 13 taxa of branching corals were recorded across four experimental plots established at Lizard Island, in the northern Great Barrier Reef. The most abundant coral was what appeared to be *Pocillopora damicornis* (but see Schmidt-Roach et al.^63^), hereafter referred to as *P.* cf. *damicornis* which accounted for 55.8% of total habitat area (Fig. 1A). A total of 29 (34.1%) coral colonies were consumed by CoTS, though only in plots at Lizard Head, North Reef, and South Island. The proportion of coral colonies consumed by CoTS within each plot ranged from 59% (10 out of 17 colonies) at Lizard Head to 0% in the Lagoon (0 out of 15 colonies). Overall cover of branching corals declined by 42.9% from 54,492 cm^2^ on Day 0 down to 31,130 cm^2^ by Day 11, by which time all starfish had moved out of the area of experimental plots. Effects were unequally apportioned among coral taxa, whereby several species of *Acropora* (*A. secale*, *A. millepora*, *A. nasuta*, and *A. humilis*) were completely consumed by CoTS (Fig. 1A). Only 26.3% of *P.* cf. *damicornis* colonies were consumed, though several larger colonies were eaten, such that the overall cover of *P.* cf. *damicornis* declined by 37.0%.

Overall, 25 out of 29 (86.2%) of colonies consumed by *A.* cf. *solaris* were occupied by coral-dwelling damselfishes (of one or more different species), but there was no difference in rates of consumption for corals that were or were not occupied by damselfish (Fig. 1A). Moreover, coral-dwelling damselfishes were not overtly aggressive towards CoTS. In two instances, we were able to directly observe responses of coral-dwelling damselfishes as CoTS approached and began feeding on their host corals. Resident damselfishes moved closer to their host coral as the starfish approached, but then remained directly above the starfish as it proceeded to consume their host coral. In both instances, at least one of the damselfishes was observed to move to, and take refuge within, a nearby coral colony as the starfish continued to feed on their original host coral.

The proportion of colonies occupied by one or more species of coral-dwelling damselfish ranged from 73.3% (11 out of 15 colonies) in the Lagoon up to 88.2% (15 out of 17 colonies) at Lizard Head. All damselfish species exhibited significant habitat selectivity at Day 0 (Fig. 1), using different corals disproportionately to their availability (based on combined projected area of all live corals of each type). *Dascyllus aruanus* was found exclusively on Pocilloporidae corals, predominantly *P.* cf. *damicornis* (91,118 individuals), but also *Stylophora pistillata* and *Seriatopora hystrix* (Fig. 1B). *Dascyllus reticulatus* was found mostly on *P.* cf. *damicornis* (111/197 individuals), *S. pistillata* and *P. meandrina*, but was also occasionally found on *Acropora* corals (Fig. 1C). *Pomacentrus moluccensis* used the greatest variety of different coral taxa, including branching *Porites* and branching *Echinopora*, which they used in far greater proportions to their availability. While there was definite redistribution of all fishes among available coral hosts following the consumption of some corals by CoTS (described below), there was no significant change in the habitat associations of *D. aruanus* during the course of this study (Fig. 1B), which continued to be found mainly living on *P.* cf. *damicornis*. However, for *D. reticulatus* and *P. moluccensis* there were significant changes in proportional use of different habitats, reflecting changes in the absolute relative abundance of different corals (Fig. 1). Most notably abundance of both pomacentrid species declined on *P.* cf. *damicornis*, although there were also smaller increases in the use of *P. meandrina* corals by *D reticulatus* and branching *Echinopora* by *P. moluccensis* that contributed to shifts in coral use.

**Figure 2:**
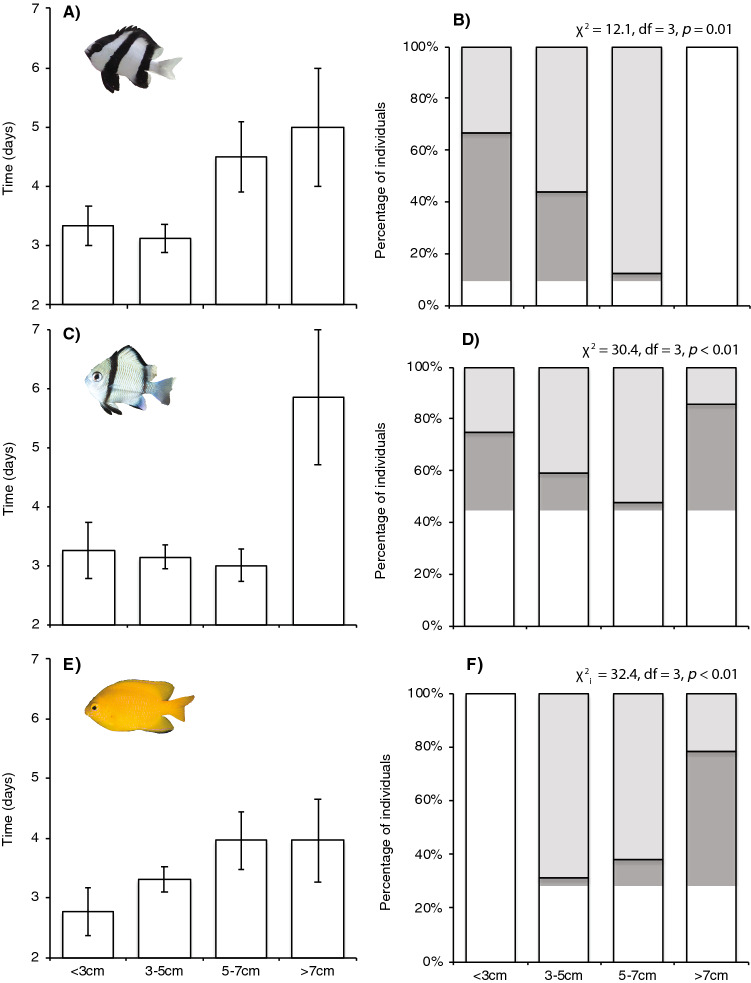
Size-dependent responses of (**A**,**B**) *Dascyllas aruanus*, (**C**,**D**) *Dascyllus reticulatus*, and (**E**,**F**) *Pomacentrus moluccensis* to mortality of host coral colonies following the introduction of crown-of-thorns starfish to experimental plots. Persistence (**A**,**C**,**E**) on dead coral hosts (consumed by crown-of-thorns starfish) is measured based on the mean (± SE) time in days that individual fishes remained on coral hosts devoid of any live tissue, which was analyzed using ANOVA (Table 1). The fate of fishes that ultimately vacated dead coral hosts (**B**,**D**,**F**) is expressed as the ratio of fishes that relocated and recolonized alternative corals hosts within the 100 m^2^ of experimental plots (expressed as a percentage of the total number of fishes displaced from coral colonies that died). χ^2^ statistics (log-linear statistic) compared frequencies of fish that moved (grey bars) versus disappeared (white bars) within each size class for each damselfish species separately.

Coral-dwelling damselfishes quickly disappeared from corals consumed by CoTS; The majority (165/182) of fishes persisted for less than 5 days on dead corals. Persistence on dead corals did not vary among the three species of damselfishes, but did vary consistently with body size (Table 1) whereby larger fishes persisted longer on dead colonies compared to small-bodied conspecifics (Fig. 2). Damselfishes were not tagged or individually recognizable, but the fate of displaced fishes was inferred based on changes in the abundance and size structure of damselfishes colonizing other coral hosts. In all, 85 (46.7%) of fishes that were displaced (initially living on corals that were thereafter consumed by CoTS) were recorded living on alternative coral hosts within the area of experimental plots. Notably, fishes only ever moved to coral hosts already occupied by other coral-dwelling damselfishes, so there was no change in the extent of diversity of different coral hosts occupied by these damselfish. Also, no fishes were ever recorded on corals immediately beyond that perimeter of the experimental plots (an experimental buffer zone) that were cleared of damselfishes at the start of the study. The proportion of displaced damselfishes that recolonized alternative coral hosts ranged from 41.1% for *D. reticulatus* up to 52.8% for *D. aruanus*, but was consistently higher for the intermediate (3–5 and 5–7 cm) size-classes compared to small (< 3 cm) or large (> 7 cm TL) individuals (Fig. 2). The size structure of fish assemblages was also very consistent among locations, except for *P. moluccensis* (Χ^2^ = 22.88, df = 9, p < 0.01), for which smaller (< 3 cm TL) juveniles were under-represented at Lizard Head.

**Table 1:** ANOVA of persistence (time in days that fishes remained on coral colonies after they were consumed by crown-of-thorns starfish), testing for differences among species (*D. aruanus*, *D. reticulatus,* and *P. moluccensis*) and size (TL: < 3 cm, 3–5 cm, 5–7 cm, and > 7 cm) of damselfishes.

Effect	SS	df	F	*p*
Species	3.3	2	0.44	0.64
Size	40.2	3	3.55	0.01
Species × size	37.4	6	1.65	0.13
Error	641.6	170		

Model used a gamma distribution, owing to a non-random distribution of the response variable.

**Figure 3:**
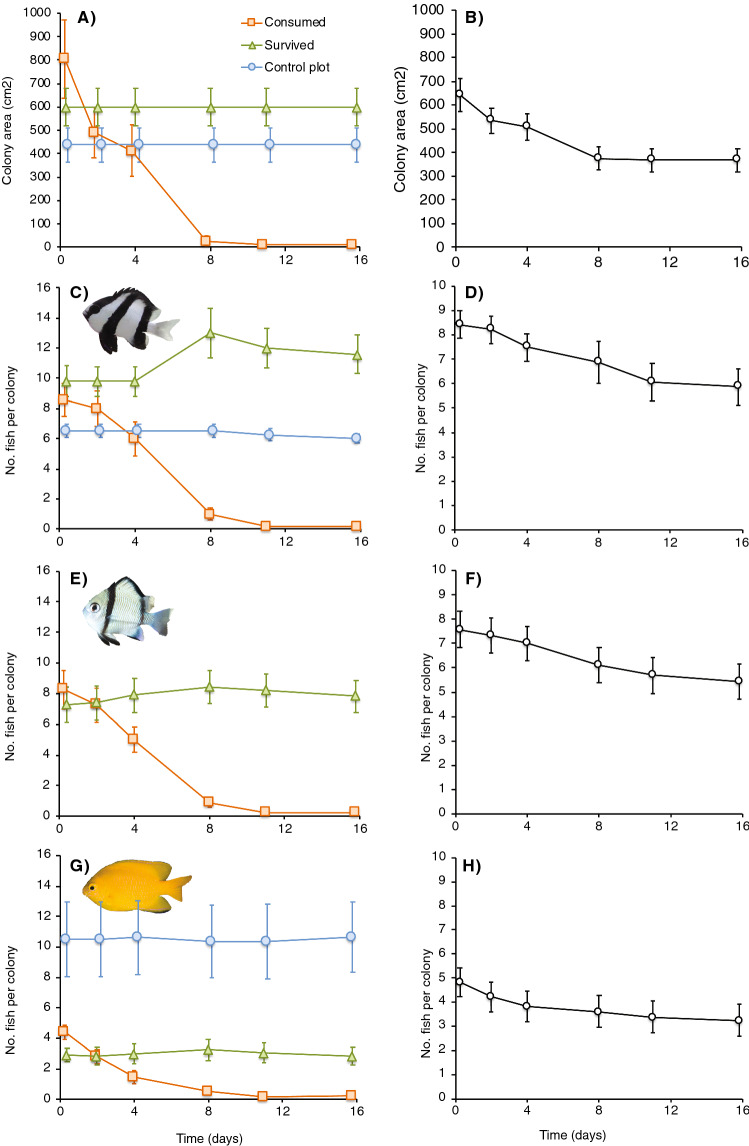
Temporal variation in (**A**,**B**) areal extent of live tissue across all branching corals and **C**–**H**) mean number of fishes per colony (± SE). Data is presented separately (in **A**,**C**,**E**,**G**) for colonies that died (consumed; red lines) versus survived (green lines) in experimental plots where starfish persisted and consumed at least some of the corals, and for the Lagoon plot (control; blue lines) where there was no mortality of branching corals throughout the study. All data is then aggregated (in **B**,**D**,**F**,**H**) to test for overall changes in habitat availability and density of fishes, as per separate analyses in Table 2.

Despite movement and recolonization of alternative coral hosts by a significant portion of displaced fishes, the overall abundance of damselfishes declined by 30.2% during the course of this study, ranging from 26.3% (31 out of 118 individuals) for *D. aruanus* to 32.9% (97 out of 295 individuals) for *P. moluccensis* (Fig. 3). All declines were directly attributable to the loss of coral hosts, consumed by CoTS, whereby all fishes that were living on these colonies moved or disappeared within 11 days (Fig. 3). This was reflected in significant changes in the abundance of fishes on corals that were consumed, whereas there was no significant change in the number of fishes associated with surviving coral colonies (Table 2; Fig. 3). There was evidence of slight increases in the number of *D. aruanus* and *D. reticulatus* on surviving colonies, reflecting the successful movement and recolonization of alternative corals hosts (Fig. 3). However, this only partially offset displacement of fishes from colonies that had been consumed, and initial increases in the abundance of fishes living on surviving corals were often short-lived (Fig. 3). Ultimately, there were significant declines in the abundance of all damselfish species through the course of this study (Table 2), and the net decline in the abundance of damselfishes (30.2%), closely corresponds with proportional declines in the overall abundance (34.1%) and cover (42.9%) of branching corals.

**Table 2:** Linear mixed effects models testing for temporal changes in the number of fish occupying distinct coral colonies, distinguishing between colonies that were consumed by crown-of-thorns starfish “TR (consumed)” versus survived “TR (survived)”.

Effect	Value/offset	SE	df	*p*
***D. aruanus *** **~ day × treatment + 1|colony**				
Intercept	1.75	0.91	422	0.05
Tr (consumed)	− 0.10	0.03	422	< 0.01
Tr (survived)	0.03	0.03	422	0.21
***D. aruanus *** **~ day + 1|colony**				
Intercept	1.36	0.38	424	< 0.01
Day	− 0.02	0.01	424	0.03
***D. reticulatus *** **~ day × treatment + 1|colony**				
Intercept	0.00	1.14	422	1.00
Tr (consumed)	− 0.16	0.04	422	< 0.01
Tr (survived)	0.02	0.03	422	0.55
***D. reticulatus *** **~ day + 1|colony**				
Intercept	2.30	0.50	424	< 0.01
Day	− 0.05	0.13	424	< 0.01
***P. moluccensis *** **~ day × treatment + 1|colony**				
Intercept	7.20	1.23	422	< 0.01
Tr (consumed)	− 0.19	0.03	422	< 0.01
Tr (survived)	0.01	0.03	422	0.91
***P. moluccensis *** **~ day + 1|colony**				
Intercept	3.20	0.56	424	< 0.01
Day	− 0.06	0.01	424	< 0.01

Separate models were also run to test for overall changes in abundance of fishes regardless of the fate of their original host colonies.

## Discussion

Population irruptions of CoTS represent the most significant biological disturbance on tropical coral reefs, killing up to 90% of corals^24,26^, which can have substantial flow on effects for coral reef fishes and especially highly specialized species that rely on corals for food, shelter or settlement^12,15,38^. However, effects of CoTS are (like many disturbances) often very patchy^61^, due partly to avoidance of corals that are actively defended by infaunal and associated organisms^30,32,33^. In this study, we did not observe any overt aggression towards CoTS by any coral-dwelling damselfishes, and there was no effect of damselfish occupation on whether or not corals were consumed. Rather, coral colonies used by coral-dwelling damselfishes were vulnerable to predation by *A.* cf. *solaris*.

All three damselfish species (*D. aruanus*, *D. reticulatus*, and *P. moluccensis*) are considered to be dependent on live coral hosts, because (i) they are almost invariably found living in close association with live corals^52,53^ and (ii) decline in abundance following localized mortality of host corals^12,15,64^. However, the specific timing and proximate mechanisms leading to declines in the abundance of these species (and many other coral-dwelling damselfishes), following acute coral loss, are largely unknown. Notably, there are reports of coral-dwelling damselfishes living on dead coral hosts for protracted periods^65,66^. Sano et al.^65^, for example, recorded both *D. aruanus* and *P. moluccensis* living on dead coral colonies several months after extensive coral mortality caused by severe outbreaks of *A. cf. solaris* in Japan. Sano et al.^65^ concluded that the abundance of damselfishes living on coral colonies is generally unaffected by the loss of live coral tissue, and only declines when physical structure of host colonies is compromised. In contrast, we found that coral-dwelling damselfishes vacated coral hosts that had been consumed by CoTS within 12 days, disappearing (moving and/or dying) long before any changes in the physical integrity of the coral skeletons. Similarly, Coker et al.^58^ showed that *D. aruanus* rapidly vacate dead (but not bleached) coral habitats when alternative coral hosts are available. The extent to which coral-dwelling damselfishes persist on dead coral may therefore, depend on the availability of alternative coral hosts, which can be very limited following severe disturbances and in highly degraded reef environments^47,65^. Alternatively, differences in the persistence of coral-dwelling damselfishes on dead coral hosts may be attributable to changes in the abundance of predators, many of which are also vulnerable to extensive coral loss and habitat degradation^41,67^, such that fishes living in highly disturbed environments may actually experience much lower risk of predation.

There is increasing evidence that coral-dwelling fishes can and will move among alternative coral hosts^58^, especially following host coral mortality. Coker et al.^58^ tagged individuals of *D. aruanus* and explicitly showed that fishes moved from corals that were experimentally bleached (and died) to nearby (1–2 m apart) healthy corals. In this study, we directly observed some damselfishes moving to very nearby corals (< 1 m apart) as soon as CoTS began feeding on their original host coral. Overall, 46.7% (85 out of 182 individuals) of displaced fishes were recorded to re-colonize other nearby occupied coral hosts, adding to the number of fishes already established within these colonies. This capacity of coral dependent fishes to move between alternative coral hosts would be expected to confer increased resilience to disturbances, especially during moderate disturbances that cause limited coral loss^45^. However, displaced coral-dwelling damselfishes only ever took up residence in coral colonies that were already occupied by other coral-dwelling damselfishes, and mainly conspecifics. This is consistent with earlier findings that coral-dwelling damselfishes (specifically, *Dascyllus* spp.) preferentially colonize coral hosts that are already occupied by conspecifics^58,68,69^ and may actually cue in on the presence of conspecifics to identify suitable microhabitats. It is also rare to find solitary individuals of coral-dwelling damselfishes within any given coral colony. In this study for example, the mean number of damselfishes (all species) found in occupied corals was 8.17 (± 1.1SE) and only 3 (out of 70) corals were occupied by a single damselfish, and always *P. moluccensis*. This suggests that there are significant benefits for group-living by coral-dwelling damselfishes^45,70^, which outweigh potential costs associated with joining established colonies or colonizing new and unoccupied coral hosts^71^. It is also possible that the few colonies (15 out of 85) of branching corals that were not already colonized by coral-dwelling damselfishes may be unsuitable as habitat for these fishes, as specialist coral-dwelling damselfish are not prepared to use alternative coral species even during a scarcity of coral hosts^15^. Even among the colonies of coral species that these species do normally use, unoccupied colonies may be unsuitable owing to their specific size or morphology^72,73^. We could not see any obvious differences between coral colonies that were and were not occupied by coral-dwelling damselfishes, though we did not explicitly quantify differences in habitat structure between these corals.

The downside of moving to coral colonies that are already occupied by conspecifics is that displaced fishes must compete with established individuals to gain access to potentially limited refuge space^74,75^, reproductive opportunities^6,76^, and food resources^71^. Our observations show that movement and recolonization success is strongly size-dependent, being lowest for both the largest (> 7 cm TL) and smallest (< 3 cm TL) size classes. Notably, larger individuals of all three fish species tended to persist on recently dead corals much longer than smaller individuals, and larger individuals were only rarely detected on alternative host corals within the area of experimental quadrats, and never for *D. aruanus* (Fig. 2). These observations might be explained by socially mediated differences in recolonization success^6,45,77^. Extensive research on the sociality and reproductive system of *D. aruanus* has shown that this species is a protogynous hermaphrodite with a hierarchical polygynous mating system^6^ and strong size-based competitive rankings^6,71^. While the number of males can vary with the size of the colony^6^, it is likely that additional males will pose a direct threat to the reproductive output of established males, and are therefore, likely to be competitively excluded from joining established colonies. Alternatively, it may be that the spatial extent of movement by displaced fishes scales with body size, such that when larger individuals did eventually vacate their dead coral hosts they may have moved well outside of the experimental plots and even beyond the limited extent of our buffer zones. In attempts to relocate *Dascyllus* to experimental colonies, Sweatman^69^ recorded movement of these fishes between coral colonies separated by > 20 m, suggesting that these fishes are certainly capable of moving well outside of our experimental plots and buffer zones.

Explanations for the low probability of very small damselfishes successfully moving and recolonizing alternative colonies following the mortality (consumption) of their original coral hosts is likely to be very different from that of larger individuals. Most notably, smaller individuals are likely to be much more readily accepted into established colonies^45^, whereas they are also likely to be much more susceptible to predation^70,78,79^. Small coral-dwelling damselfishes (< 3 cm TL) are more closely associated with specific coral growth forms than larger conspecifics^12,80^ and likely to be very vulnerable to predation while they remain on recently dead coral colonies^51^, potentially explaining why we did not record any such damselfishes living on dead colonies for > 4 days. However, these individual are also likely to be very vulnerable to predation when trying to move to alternative coral hosts^78^, thereby limiting their opportunity to try and integrate into established groups of coral-dwelling damselfish on alternative coral hosts.

Despite the movement of damselfishes and successful recolonization of alternative corals hosts, especially among medium sized individuals, there were net declines in the abundance of all damselfish species (*D. aruanus*, *D. reticulatus*, and *P. moluccensis*) through the course of this study. Moreover, net declines in the abundance of fishes were concordant with levels of host coral mortality, as recorded previously for many coral-dwelling damselfishes^12,64,81^. This suggests that the movement and redistribution of coral-dwelling species in responses to changes in the abundance and distribution of coral hosts does little to mediate longer-term effects of habitat loss. Observed declines in the abundance of these fishes are attributable to emigration (extensive movements beyond the immediate area encompassed by experimental plots and buffer zone) and/or mortality of fishes that persisted on dead coral hosts or attempted to move to alternative coral hosts. Given that no displaced fishes were relocated within suitable coral hosts in the buffer zone (surrounding each experimental plot), we initially assumed that successful movement and recolonization of fishes to alternative coral hosts is likely to be spatially restricted. However, the effectiveness of the buffer zone for detecting fishes moving outside of experimental quadrats has to be questioned given that fishes never relocated to colonies that were not already occupied by conspecifics. Even so, we did record at least some displaced fishes (46.7%) recolonizing alternative coral hosts within the experimental plots. The fact these movements did not buffer against overall coral loss is attributable to limited persistence of elevated densities of coral-dwelling fishes on these surviving coral colonies, possibly reflecting inherent limits on the number of fishes that can be sustained within individual corals^10,74,82^. Given our inability to distinguish individual fishes of the same size we do not know whether fishes that moved ultimately succumbed, or whether these individuals effectively displaced other fishes of equivalent size^45^.

In conclusion, this study shows that the coral-dwelling damselfishes, *D. aruanus*, *D. reticulatus* and *P. moluccensis*, generally avoid associating with dead coral hosts, but have some capacity to move and colonize alternative coral hosts following complete mortality of previous coral hosts. However, the capacity to recolonize alternative coral hosts does not necessarily confer increased resilience for populations of coral-dependent species, which are subject to increasing incidence, severity and diversity of disturbances that cause host coral mortality^39^. Rather, local abundance of coral-dwelling damselfishes declined in approximate accordance with proportional loss of suitable coral habitat^12,15^, whereby coral-dwelling fishes failed to expand the range of coral habitats used following habitat depletion, and there was limited capacity to sustain higher densities of damselfishes on already occupied coral hosts^10,74^. Moreover, very severe and large-scale disturbances caused by anthropogenic climate change, will increase the extent of coral loss^22^ and have disproportionate impacts on branching corals^83^. Highly specialized fishes with specific reliance on live corals for food or habitat are therefore, extremely vulnerable to sustained and ongoing changes in the condition and structure of coral reef ecosystems, as has been suggested previously^11,12,40,82,84,85^.

## Materials and methods

All research was conducted in accordance with the James Cook University ethics and research integrity guidelines, and with explicit approval by the Great Barrier Reef Marine Park Authority (qualifying as limited impact research) as well as James Cook University Animal Ethics Committee.

### Experimental setup

This study was conducted at Lizard Island (14° 40′ S, 145° 27′ E), on the northern Great Barrier Reef, Australia; 10 × 10 m (100 m^2^) experimental plots were established at each of four different locations (North Reef, South Island, Lizard Head and Lagoon). All plots were established at the base of the reef slope (7–10 m depth) in areas with a rubble substrate where isolated colonies provided relatively independent units of habitat. To explore the responses of these fishes to host coral mortality, this study took advantage of high local densities of crown-of-thorns starfish, which naturally consumed corals within the immediate area of experimental plots. The four 100 m^2^ experimental plots together encompassed a total of 85 coral colonies, most of which (71/85 colonies or 83%) were occupied by one or more species of the coral-dwelling damselfishes, *Dascyllus aruanus*, *D. reticulatus*, and/or *Pomacentrus moluccensis*. Sites were selected where there were abundant CoTS (4–12 starfish per plot), but no apparent recent coral mortality. However, *A.* cf. *solaris* are only very occasionally observed within the lagoon^86^ and did not consume any corals within the experimental plot established at this location, effectively serving as a ‘control’. Because CoTS do not directly affect the physical structure of coral colonies^39^, and natural erosion and decomposition of dead coral colonies takes years rather than days^40^, changes in the condition of coral habitats were solely affected through the loss of live tissue with little or no change in physical integrity of coral hosts.

To account for localized movement of damselfishes outside of the experimental plot we cleared a 5-m wide buffer zone (a total of 300 m^2^) around each of the plots, removing all damselfishes from within live coral colonies (using clove oil and hand nets) but leaving the now vacant coral colonies in place. Without having tagged all individual damselfishes, there was limited capacity to assess long-distance movement of displaced damselfishes, but it was anticipated that if damselfishes moved outside of the experimental plot, at least some individuals would be found within the 5 m buffer zone around each plot. If fish suffered mortality with the death of their host colony, this would be apparent from a net decline in the abundance of fish in each plot. If however, fishes moved between coral colonies within each plot, then the total abundance and size structure of fish within each plot would remain constant despite a decline in the number of live coral colonies.

In order to document responses of coral-dwelling damselfishes to host coral mortality, the distribution and abundance of coral-dwelling damselfishes within each experimental plot and associated buffer zones was recorded immediately before (on day 0), and then every 2–5 days until there were no coral-dwelling damselfishes remaining on coral colonies that had been consumed by *A.* cf. *solaris*, which occurred on Day 16. Every damselfish sheltering in each coral colony was identified to species and its size (total length) visually estimated to the nearest cm. During surveys, two divers would independently count and recount all damselfishes in each successive coral colony, until they arrived at a consensus. For every coral colony, the maximum diameter and perpendicular diameter were recorded, from which we calculated 2-dimensional projected area^87^. Surveys of corals and coral-dwelling fishes took up to 80 min per plot and all four plots were surveyed within the same day.

### Data analyses

To test whether *A.* cf. *solaris* avoided coral colonies that were occupied by coral-dwelling damselfishes, we compared the ratio of colonies consumed versus those that were not consumed (restricting comparisons to the three experimental plots where at least one coral was consumed) for colonies that were and were not occupied by one or more species of damselfish, using the Pearson statistic with Yate’s correction. It was not possible to take full account of taxonomic differences in coral hosts and data was pooled across all coral species to maximize cell counts. However, it was recognized that these comparisons may be confounded by inherent feeding preferences of *Acanthaster* spp.^29^, especially if starfish preferentially target coral species that are generally not used by coral-dwelling damselfish (e.g., digitate *Acropora* spp.). Therefore, a separate comparison was also conducted based on the single most abundant coral taxa, *Pocillopora* cf. *damicornis*, which was used by all three species of damselfish.

To explore the differential availability coral species, and occupation of these potential coral hosts by each of the three coral-dwelling fishes (*D. aruanus*, *D. reticulatus*, and/or *P. moluccensis*), the 2-dimensional planar area of live tissue for each coral colony was calculated from measurements of external dimensions less proportional area of partial mortality. Initial habitat preferences were analyzed by comparing the relative use of different coral taxa (total frequencies) by each damselfish species to their proportional availability on day 0. Pearson statistics were used to take account of all coral types that were and were not used. Separate analyses were conducted for each species of damselfish, combining data from all four plots and locations (Lizard Head, North Reef, South Island and Lagoon). Changes in habitat use thereafter, were analyzed using the log-likelihood statistic to compare frequencies of each damselfish across the limited range of different coral taxa that were used by each species of damselfish on day 0 versus day 16, following Manly et al.^88^. These analyses (treating each individual fish independently) are potentially confounded by aggregative behavior of coral-dwelling damselfish^59^, though tests of habitat preferences explicitly account for the areal extent (rather than number of distinct colonies) of each coral type. Moreover, simple comparisons of occupied versus unoccupied colonies would obscure individual differences in recolonization success and subsequent habitat-associations of fishes.

To test for taxonomic and size-based differences in the responses of damselfishes to host coral mortality, we compared time (in days) that individual fishes persisted on dead coral hosts, which had been consumed by *A.* cf. *solaris*. Persistence of fishes on dead corals was estimated based on the maximum duration between death of the host coral and the first observation in which fishes were absent, e.g., even if fishes vacated coral hosts within hours of the colonies being consumed, persistence would be estimated to be two days given that was the minimum duration between observations. This was analyzed using GLM with a gamma distribution, given that the response variable (time) was non-negative and far from normally distributed (Shapiro–Wilk test; W = 0.68, p < 0.01). Secondly, we tested for size-specific differences in the fate of displaced fishes, comparing the number of displaced fishes (formerly living on coral hosts that were consumed by starfish) that did versus did not successfully recolonize other corals within experimental plots using the log-linear statistic. Since no fish were observed within the buffer zone, we assumed that those fish that disappeared from corals consumed by *A.* cf. *solaris*, either moved well outside the experimental plots and buffer zones, or died. For frequency analyses, fish were assigned to one of four different size classes (< 3 cm, 3–5 cm, 5–7 cm, > 7 cm).

Changes in the overall abundance of each damselfish species on corals, were analyzed through the course of the experiment (16 days) using a repeated-measures linear mixed-effects models in “nlme”^89^. Individual host corals were used as the random effect, effectively testing for changes through time in the number of fish per host coral. Models were initially run accounting for the fate of colonies, distinguishing between those individual coral colonies that died versus survived, as well as treating all colonies in the control plot separately. This revealed whether there were temporal changes in the number of fishes on corals that survived, which may be caused by colonization of fishes that vacated dead coral hosts. We also tested for overall changes in the abundance of damselfish (each species separately), thereby assessing whether movement and recolonization of surviving coral provided resilience to coral loss. All analyses were conducted in R 3.3.2^90^.
